# Multifunctional Quantum Sensing Using Single Spins in a van der Waals Crystal

**DOI:** 10.1002/advs.77559

**Published:** 2026-09-06

**Authors:** James Liddle‐Wesolowski, Konosuke Shimazaki, Jiyun Kim, Benjamin Whitefield, Kenji Watanabe, Takashi Taniguchi, Mehran Kianinia, Igor Aharonovich

**Affiliations:** ^1^ School of Mathematical and Physical Sciences University of Technology Sydney Ultimo New South Wales Australia; ^2^ ARC Centre of Excellence for Transformative Meta‐Optical Systems University of Technology Sydney Ultimo New South Wales Australia; ^3^ Research Center for Electronic and Optical Materials National Institute for Materials Science Tsukuba Japan; ^4^ Research Center for Materials Nanoarchitectonics National Institute for Materials Science Tsukuba Japan

**Keywords:** dual sensing, hBN, magnetometry, ODMR, thermometry, ZPL

## Abstract

Nanoscale thermometry and magnetometry are in high demand across a wide range of scientific and technological applications. In this context, optically addressable spins in solids have emerged at the forefront of on‐chip quantum sensing. However, simultaneous quantum sensing of multiple parameters (e.g., temperature and magnetic field) using the same spin sensor remains challenging due to cross‐sensitivity to multiple physical quantities. Here, we demonstrate independent dual sensing of temperature and magnetic field using spin‐1/2 transitions within the spin complex quantum emitters in hexagonal boron nitride (hBN)). We experimentally verify the independent response of the zero‐phonon line (ZPL) position to temperature and of optically detected magnetic resonance (ODMR) to magnetic fields. Furthermore, we demonstrate local temperature sensing of a microcircuit while simultaneously measuring an external magnetic field. Our results establish quantum emitters in hBN as a robust platform for multifunctional quantum sensing under realistic operating conditions.

## Graphical abstract

The work demonstrates a dual quantum sensing of temperature and magnetic field using spin‐complex quantum emitters in hexagonal boron nitride (hBN). Temperature sensing via zero‐phonon‐line shifts and magnetometry via optically detected magnetic resonances, enable simultaneous local thermometry and magnetic‐field measurements. These efforts establish hBN as a robust multifunctional quantum‐sensing platform.

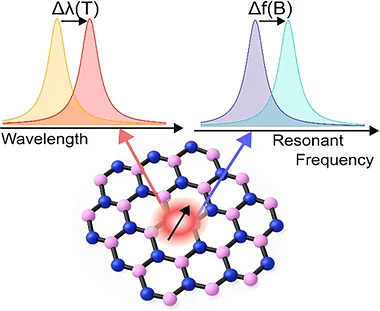

## 1 Introduction

Nanoscale quantum sensing is becoming an important aspect of many applications spanning chemical reactions, bio‐imaging, and nanoelectronics [1, 2, 3, 4]. Amongst a variety of current sensing techniques [5, 6, 7, 8, 9], solid‐state quantum emitters with optically addressable spins have attracted significant attention. These defects are often hosted by solid‐state materials, which makes them compatible with a wide variety of applications [10, 11, 12].

Amongst the variety of color center hosts, defects in diamond, silicon carbide, and hexagonal boron nitride (hBN) have been extensively studied [13, 14, 15, 16, 17, 18, 19, 20, 21]. A subset of defects in these hosts possesses a triplet ground state and exhibits a clear optically detected magnetic resonance (ODMR) signature. Consequently, the ODMR transitions can be utilized to sense external magnetic fields, electric fields, pressure, or temperature [12, 22, 23, 24]. However, simultaneous sensing using the same quantum sensor is impractical, requiring complex calibrations and measurement procedures [25, 26, 27, 28].

To address this challenge, one requires a narrowband quantum emitter that reacts independently to temperature and magnetic field. This can be achieved by monitoring the shift in the zero phonon line (ZPL) to gain information about the temperature, while independently monitoring the spin resonance to measure the magnetic field. Here, we demonstrate that such a system exists in hBN, leveraging a spin‐1/2 resonance.

**FIGURE 1:**
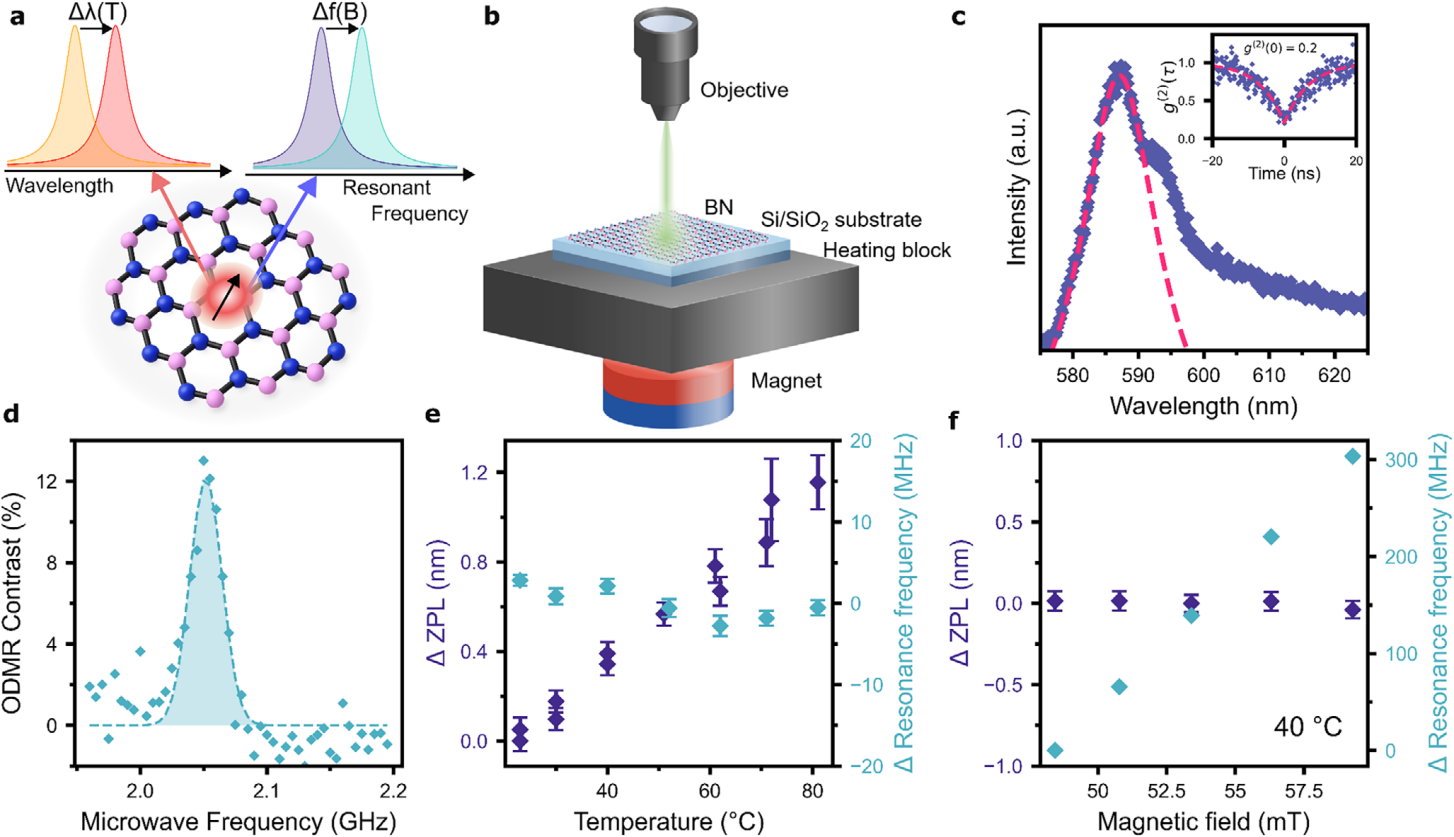
Concept demonstration of simultaneous sensing of temperature and magnetic field using a quantum emitter in hBN. (a) A graphic depiction of a temperature‐dependent PL spectra and a magnetic field‐dependent ODMR resonant frequency. (b) Sample stage schematic with labeled components. (c) The PL spectra of the emitter marked in Figure S1, the emission has a wavelength of 587 nm and a ∼15 nm FWHM. Inset: Autocorrelation of PL emission, with a g2(0) = 0.2. (d) The ODMR contrast for the emitter in (c) the spectrum has a contrast of ∼11%, with a peak position of ∼2.05 GHz. (e) Change in ZPL position (purple) and resonant frequency (cyan) as a function of temperature on the heating block. (f) Change in ZPL position (purple) and in resonant frequency (cyan) as a function of an external magnetic field, recorded at 40°C on the heating block.

Figure 1a illustrates the concept of dual sensing of magnetic field and temperature by combining ODMR and ZPL shifts from a single quantum emitter in hBN. The magnetometry system implemented in this work is controlled by the spin Hamiltonian of the optically active defect. For a typical spin‐1 system, the spin Hamiltonian is shown in Equation (1).

(1) $$\hat{H}=D\left(\hat{S_{z}^{2}}\;-\;\frac{1}{3}S\left(S+1\right)\right)+E\left(\hat{S_{x}^{2}}-\hat{S_{y}^{2}}\right)+g\mu_{B}B\cdot \hat{S}$$

Where D and E are zero‐field splitting (ZFS) parameters that come from the internal electron interaction and the structure of the lattice. S is the spin angular momentum, *g* is the electron g‐factor taken at ∼2, 𝜇_B_ is the Bohr magneton and B is the applied magnetic field. The final term in Equation (1) is the Zeeman interaction, which describes the coupling of an external magnetic field to the spin. As the ZFS parameters are dependent on the lattice structure, any thermal expansion of the lattice directly changes the resonance frequency. For spin‐1 systems, a temperature driven change in the ZFS parameters will produce a resonance frequency shift that can be confused with a change in the applied magnetic field.

On the other hand, for a spin‐1/2 system, there is no ZFS interaction, and the Hamiltonian is reduced to only the Zeeman interaction (Equation (2)).

(2) $$\hat{H_{Zee}}=g\mu_{B}B\cdot \hat{S}$$

The two spin states *m_s_*  =   ± 1/2 split linearly with applied magnetic field, resulting in an ODMR resonance frequency that changes at the rate of the electron gyromagnetic ratio (∼28 MHz /mT) [29]. Without any contributions from the ZFS, the ODMR resonance frequency is temperature independent, making the spin‐1/2 system ideal for magnetometry [30, 31, 32, 33, 34].

The thermometry sensing is based on the redshift of the ZPL position that is caused by the temperature dependence of the electronic bandgap. The ZPL wavelength shift is insensitive to Zeeman interactions created by the magnetic field [35]. By exploiting these two independent parameters, simultaneous sensing of the magnetic field and temperature can be achieved using a single quantum emitter.

To test the multifunctional sensing capability, an hBN flake hosting a variety of ODMR active quantum emitters (see Methods for fabrication details) is placed on a heating block with a position‐controlled magnet. The setup is shown schematically in Figure 1b.

Figure 1c shows a quantum emitter with a narrow emission peak. The inset includes the second‐order correlation function (g^(2)^(*τ*)), confirming that the emitter is a single‐photon source. The emitter also exhibits an ODMR signal, Figure 1d. Based on the resonance frequency of ∼2.05 GHz, the magnetic field was calculated as ∼73mT, recorded at 40°C. The DC sensitivity of this emitter is ∼4.7 µT/√Hz. A confocal photoluminescence (PL) map of the emitter is included in Figure S1.

Next, the temperature‐dependent ZPL peak position and ODMR resonance frequency of the emitter were characterized, as shown in Figure 1e. With increasing temperature from 23°C to 80°C, the ZPL exhibited a redshift with a thermal response of 0.019 nm/°C (purple data, Figure 1e). This thermal shift is comparable to the previous study [36]. In contrast, the ODMR resonance frequency remains nearly constant over the same temperature range, with a standard deviation of ∼ 2.0 MHz, as shown in cyan in Figure 1e. The heating block temperature was measured using a thermal couple.

The independence of the ZPL position from the magnetic field was verified by recording emission spectra under varying magnetic fields, as shown in Figure 1f. Shifting the magnet in 0.5 mm steps over a 2 mm range, the ODMR resonance frequency was changed by ∼300 MHz, corresponding to a magnetic field change of ∼10.7 mT. This value agrees with the simulated magnetic field change of 10.9 mT (Figure S2). On the other hand, the ZPL peak position remained stable with a standard deviation of ∼ 0.02 nm. These measurements were performed at a temperature of 40°C to demonstrate that both ZPL and ODMR signals can be read out simultaneously under the modified temperature and magnetic field. A corresponding room temperature dataset is provided in Figure S3. Combined, these results validate the independent responses of the ZPL and ODMR to temperature and magnetic field, enabling simultaneous dual sensing with a single emitter.

**FIGURE 2:**
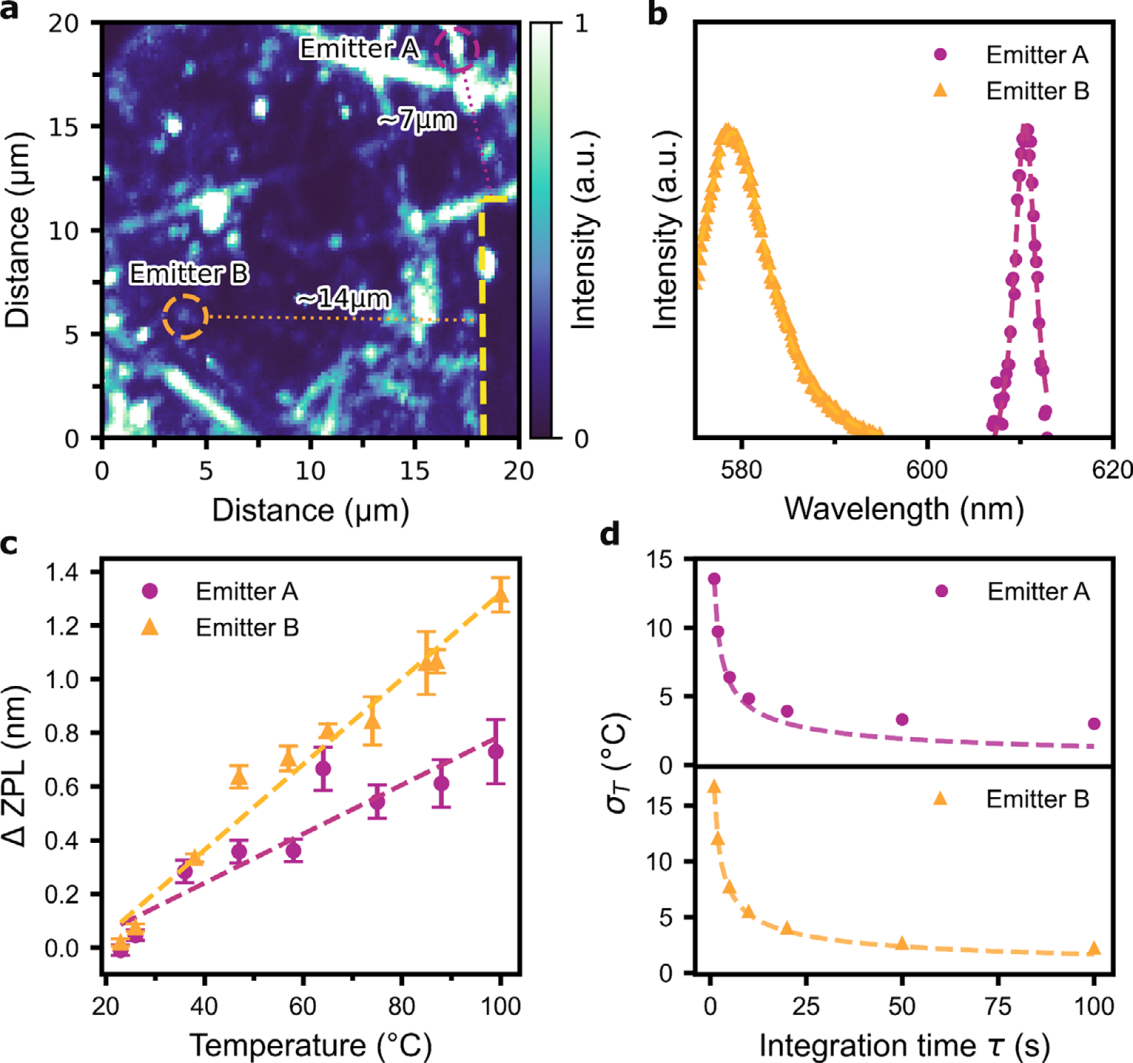
Characterization of emitters used for simultaneous sensing. (a) Normalized PL map with hBN flake edge marked in yellow, Emitters A (magenta circle) and B (orange triangle) are marked with a circle. The area enclosed by the yellow lines is the location of the circuit. The distances from the wire to Emitter A and B are printed on the map and are ∼7 and ∼14 µm, respectively. (b) PL emission spectra for Emitters A (B) located at wavelengths 610 nm (579 nm), with a FWHM of ∼2.8 nm (∼9.6 nm). (c) The change in ZPL position as a function of temperature on the heating block for Emitters A and B, with linear fits of 0.0092 nm/°C and 0.016 nm/°C, respectively. (d) Temperature uncertainty of the thermometer as a function of integration time. Dashed curve is a fitting curve of the shot noise limit $1/\sqrt{N_{\mathrm{ph}\;}}$.

To demonstrate the simultaneous temperature and magnetic field capabilities, two optically active emitters were identified near the heating circuit. Figure 2a shows a confocal map with Emitter A and B, located at distances of ∼7 and ∼14 µm from the edge of the heating circuit, respectively (marked by a yellow dotted line). The spectra of the emitters are shown in Figure 2b. The Emitters A (and B) exhibit a different full width at half maximum (FWHM) of 2.8 nm (and 9.6 nm), which are still narrow enough to carry the high‐resolution temperature sensing. Additional optical properties of the two emitters are provided in Figure S4. The calibration curve of the temperature dependent ZPL for both emitters is shown in Figure 2c. The heating is performed using a thermal block heating element, as shown in Figure 1.

To assess the temperature sensing performance, we evaluated the uncertainty as a function of the integration time of the spectrum (Figure 2d). The uncertainties were extracted from the error of the Lorentzian fitting to the emission spectrum at each accumulation time. To extract the uncertainties, we fit the shot noise limit model $A/\sqrt{N_{ph\;}}$ to the experimental result. Where A is a fitting parameter representing sensitivity, and *N_ph_* is the photon number. As the photon number is proportional to the integration time (*t_int_*), the model can be described by $A^{\prime }/\sqrt{t_{int\;}}$. The extracted uncertainties of Emitter A and B are ∼$13.4^{\circ }C/\sqrt{Hz}$ and ∼$16.4^{\circ }C/\sqrt{Hz}$, (and for 1 min integration, the sensitivity is 1.7°C and 2.1°C), respectively. We note that these values are mainly limited by the signal‐to‐noise ratio of single‐emitter measurements compared to previous studies worked on the ensemble emitters (2.9 ± 1.3°C/$\sqrt{\mathrm{Hz}}$) [37].

**FIGURE 3:**
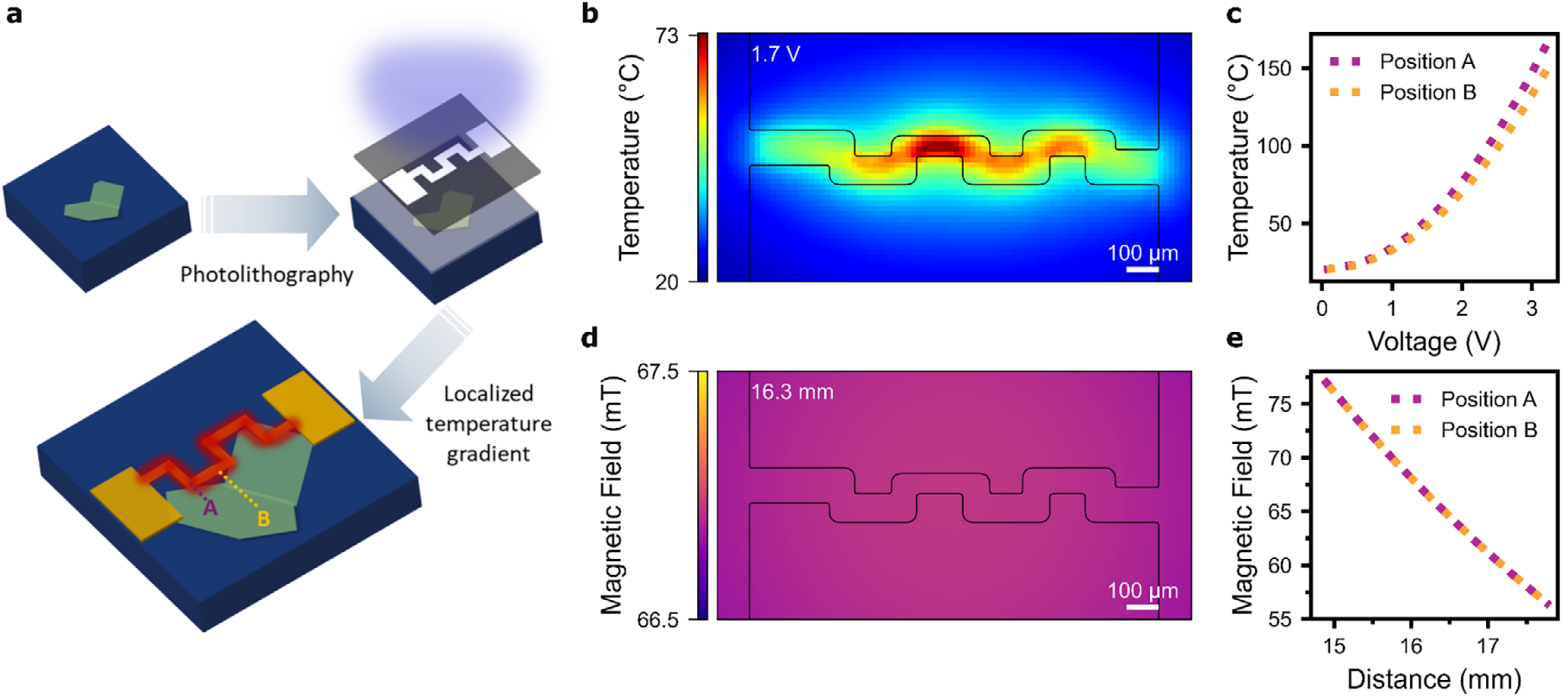
Microscale heating circuit and resulting Joule‐effect simulation. (a) Illustration of microscale heating circuit fabrication. Consisting of the flake selection followed by the photolithography step. Photoresist was spun over the substrate and was exposed to patterned UV light. A voltage was applied to the circuit and a localized temperature gradient was created. The red line indicates the heating circuit. Position A (magenta) and B (orange) indicate emitter positions closer and farther from the heating region, respectively. (b) Simulated temperature distribution of the circuit heating at 1.7 volts. (c) Simulated temperature recorded from position A (magenta) and position B (orange) at distances of 7 and 14 µm, respectively. (d) Simulated magnetic field with a magnet placed 16.3 mm underneath the substrate. (e) Simulated magnetic field taken from the same positions in (c).

We now turn to study the dual sensing modality of the quantum emitters under realistic conditions. For this purpose, we designed and fabricated a micro heating circuit to generate localized temperature variation on the emitters. The circuit was patterned near the hBN flake to facilitate controlled local temperature gradients on the emitters. A schematic of the procedure is shown in Figure 3a.

To verify that our circuit design can generate a detectable temperature gradient at micrometer scale, we performed Joule heating simulation. Figure 3b shows the simulated temperature distribution (Joule heating) using COMSOL at an applied voltage of 1.7 V. The narrowest region at the center of the wire reaches a maximum temperature of approximately 73°C, while a temperature gradient of 0.35°C/µm is observed over a distance of 50 µm from the wire edge. Figure 3c shows the voltage‐dependent temperature profile extracted from the simulation. Two representative emitter positions were defined at distances of 7 µm (position A) and 14 µm (position B) from the wire edge along the y‐direction, while centered along the x‐direction. Note that the positions A and B in the simulated result correspond to the positions of Emitter A and B from the circuit, respectively. The simulation revealed a clear temperature difference between positions A and B, confirming that the circuit generates a spatially localized temperature gradient suitable for local temperature sensing.

Unlike the temperature distribution, the magnetic field is distributed uniformly across the hBN area. Figure 3d shows the calculated magnetic field distribution when the magnet is located at the center and 16.3 mm from the substrate. The average strength of the magnetic field is ∼66.9 mT. The magnetic field intensity as a function of the distance from the magnet is shown in Figure 3e. The difference in magnetic field intensity between position A and B is below 10 µT within our observed area (<100 µm × 100 µm), suggesting that the magnetic field is uniformly applied over the sample.

**FIGURE 4:**
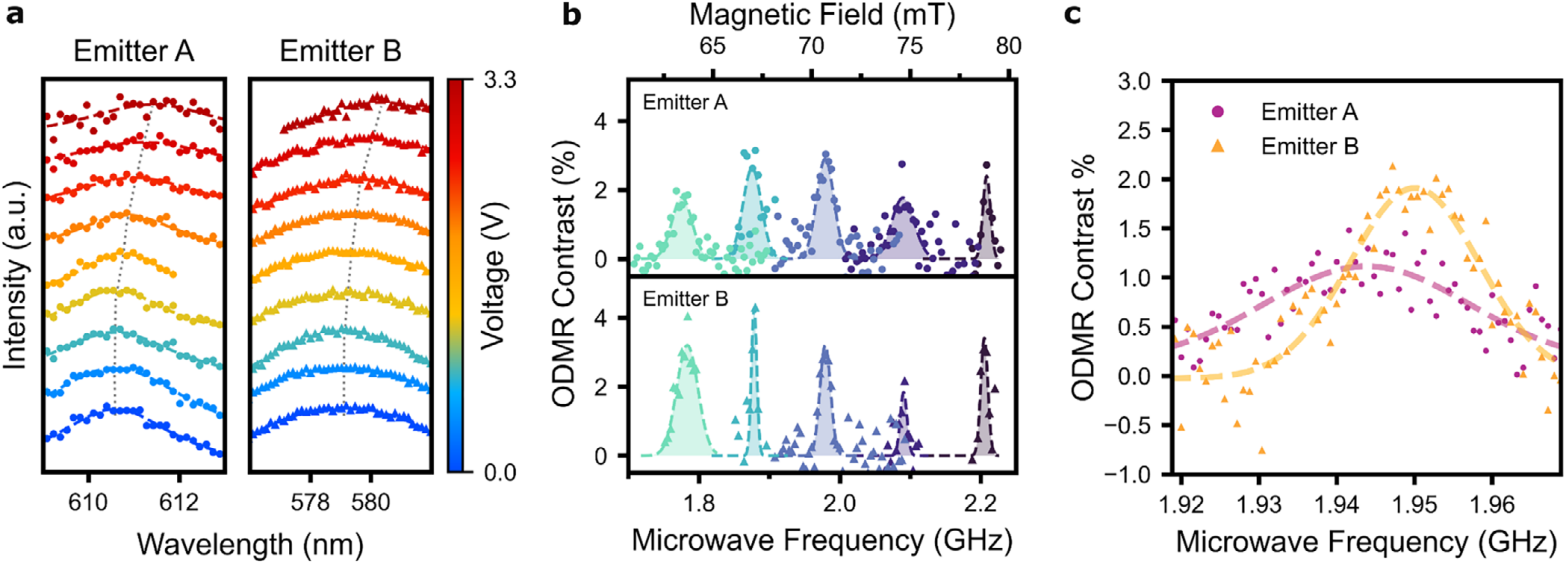
Dual sensing characteristics of Emitters A and B. (a) Emission spectra for Emitters A and B over the applied voltage range of 0 to 3.3 V. The grey line marks the location of the fitted ZPL peak positions. (b) ODMR spectra for Emitters A and B recorded at different magnetic field strengths. (c) ODMR spectra for Emitters A and B recorded with localized magnetic fields generated by randomly positioned magnetic particles.

The combined, simultaneous temperature and magnetic field sensing for Emitters A and B is shown in Figure 4. Both emitter's thermal response is depicted in Figure 4a. Over the applied voltage range of 0 to 3.3 V, Emitters A (B) experienced ZPL redshifts of ∼0.82 nm (∼1.2 nm), corresponding to a local temperature of ∼112°C (∼101°C), respectively. Figure S5a,b show the isolated temperature dependent voltage together with a comparison to the simulation presented in Figure 3c. The ODMR signal was independent from temperature with standard deviations of ∼2.9 MHz (∼1.8 MHz) across this temperature range (Figure S6a). The magnetic field response for both emitters is shown in Figure 4b. As expected, both emitters experienced similar changes in the resonant frequency under the applied magnetic field. The magnetic field magnitude at each magnet position was calculated from the measured microwave resonance frequency. The resonant frequency's dependence on the magnetic field is separately presented in Figure S5c,d, alongside a comparison to the simulation shown in Figure 3e. Emission spectra acquired at the same time confirmed the insensitivity of the ZPL emission against the applied magnetic field, with standard deviations of ∼0.07 and ∼0.06 nm for Emitters A and B, respectively (Figure S6b). The DC magnetic field sensitivity of Emitters A and B was evaluated using a theoretical sensitivity model [38].

(3) $$\eta_{DC}=P_{L}\frac{h}{g\mu_{B}}\frac{\Delta \nu }{C\sqrt{R}}$$

where 𝒫_L_ is a constant associated with the Lorentzian line shape (∼0.77), h is Planck's constant, Δν is the ODMR linewidth, C is the ODMR contrast, and R is the photon count rate. The parameters *g* and 𝜇_B_ are shared with Equations (2) and (3). The sensitivity of Emitters A and B were calculated as ∼8.3 µT/√Hz and ∼3.0 µT/√Hz, respectively.

To demonstrate the localized sensing capability of the emitters, magnetic nanoparticles were dropcasted over the flake, randomly distributing magnetic dipoles across the flake. In the presence of the nearby nanoparticles, both emitters experience a unique local magnetic field. The localized environment is reflected in the ODMR spectra (Figure 4c), where Emitter A (and B) exhibited a resonance frequency of ∼1.94 GHz (and ∼1.95 GHz), corresponding to a magnetic field with the strength of ∼69.42 mT (and ∼69.64 mT).

We demonstrated dual sensing of temperature and magnetic field simultaneously using quantum emitters in hBN. We showed that the ZPL position is insensitive to magnetic field variations, while the ODMR resonance remains stable over the investigated temperature range. This result enables independent readout of the two physical quantities without cross‐talk. The achieved sensitivities of ∼13.4°C/$\sqrt{\mathrm{Hz}}$ for thermometry, and ∼3.0 µT/$\sqrt{\mathrm{Hz}}$ for magnetometry are promising for practical, real‐world applications – especially in microelectronics. The intrinsic decoupling of temperature and magnetic field responses provides a significant advantage for practical sensing applications, where simultaneous and reliable multi‐parameter measurements are required.

Additional parameters, such as fluorescence lifetime, can enable a complementary degree of freedom to sense chemical modifications, providing an all‐around multifunctional quantum sensor. All in all, our results solidify the employment of quantum emitters in hBN as a promising platform for nanoscale sensing under realistic operating conditions.

## 2 Methods

### 2.1 Sample Preparation

Carbon‐doped hBN was exfoliated onto diced 285 nm SiO_2_/Si wafers using the scotch tape method. Visible emitters were activated by a high‐temperature oxygen anneal. Using a Lindbergh blue tube furnace, the tube was connected to a scroll pump and purged with oxygen. The furnace was heated to 1000°C with an oxygen flow rate of 1000 sccm and held for 4 hrs in this environment. The annealed substrates were placed in UV ozone for 4 hrs to remove any surface deposition and unstable emitters.

### 2.2 Fabrication of Heating Circuit

To design the micro‐scale heating circuit, photoresist (AZ‐1518) was deposited onto the prepared hBN substrate by spin‐coating. Then, the sample was placed on PLANCK maskless UV lithography and exposed through a designed pixel array (1920×1080) to pattern a micro‐scale heating circuit near the hBN flake. Then, we deposited Chromium using sputtering with a Dynavac CS 12–14 Dual Sputter System. Residual photoresist was removed by placing the sample in 40°C acetone for 1 hrs after deposition.

### 2.3 Optical Characterization

PL measurements were performed using a home‐built confocal setup that used a 532 nm CW laser and a x100 Olympus objective with a 0.9 numerical aperture. The surface of the sample was measured with a scanning mirror and a 568 nm long pass filter to block the laser falling on Excelitas avalanche photodiodes (APDs). An additional 617/14 nm band‐pass filter was used for “Emitter A” when performing autocorrelation and ODMR measurements. HBT measurements were recorded with two APDs, and coincidence was correlated by a PicoQuant time tagger.

### 2.4 Dual Temperature and Magnetic Sensing

Global heating measurements were performed using a custom‐built heating coil block, and the temperature was read out using a thermocouple and a multimeter. The power to the heating block and voltage were supplied to the heating micro circuit with a Tektronix 2623B system SourceMeter. CW ODMR was performed using an AnaPico APSIN4010 signal generator, the resulting frequencies were then amplified with a KeyLink KB0727M47C. A NdFeB magnet (N35) was placed below the sample on a z‐axis controlled stage to provide a change in magnetic field.

### 2.5 Simulation

The Joule heating of the micro‐circuit was simulated using COMSOL Multiphysics 6.3. A three‐dimensional steady‐state heat transfer model was employed. The applied current was determined from the experimentally applied voltage divided by the measured average resistance of the circuit. The temperature of the Si substrate and the surrounding air was set to 20°C. Heat dissipation to the substrate and the surrounding air was included via thermal conduction. Material properties of chromium and silicon were taken from the COMSOL material library. The current was applied to a 1 mm × 1 mm chromium pad, matching the experimental configuration.

The magnetic field intensity distribution was calculated using a numerical analysis implemented in Python. The magnetic field was computed based on the Biot–Savart law by modeling the cylindrical magnet as a stack of current loops. The axial field of each loop was described by the standard expression, and the off‐axis dependence was approximated using a distance‐based formulation:


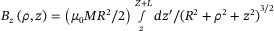

where $\rho =\sqrt{x^{2\;}+y^{2\;}}$ represents radial distance, *μ*_0_ is vacuum permeability, M is the magnetization, *R* is the radius of the magnet, *L* is the length of the magnet, and *z* is the axial distance from the observation point.

## Author Contributions

**James Liddle‐wesolowski**: investigation, writing – original draft. **Konosuke Shimazaki**: investigation, writing – original draft. **Jiyun Kim**: investigation. **Benjamin Whitefield**: investigation. **Kenji Watanabe**: investigation. **Takashi Taniguchi**: investigation. **Mehran Kianinia**: investigation, conceptualization, writing – review and editing. **Igor Aharonovich**: conceptualization, funding acquisition, writing – review and editing.

## Conflicts of Interest

The authors declare no conflicts of interest.

## Supporting information




**Supporting File**: advs77559‐sup‐0001‐SuppMat.docx.

## Data Availability

The data that support the findings of this study are available from the corresponding authors upon reasonable request.
