# Supplementary material for: Multifunctional Quantum Sensing Using Single Spins in a van der Waals Crystal
Source: Adv Sci (Weinh). 2026 Sep 6:e77559. Online ahead of print. doi: 10.1002/advs.77559 (PMC13547707; doi:10.1002/advs.77559)
Supplement: Supplementary file 1 [file ADVS-9999-e77559-s001.docx]

**Supporting Information**

**Multifunctional quantum sensing using single spins in a van der Waals crystal**

James Liddle-Wesolowski^1,2^, Konosuke Shimazaki^1,2^, Jiyun Kim^1, 2^ , Benjamin Whitefield^1,2^, Kenji Watanabe^3^, Takashi Taniguchi^4^and Mehran Kianinia^1,2^, Igor Aharonovich^1,2^

^1^ School of Mathematical and Physical Sciences, University of Technology Sydney, Ultimo, New South Wales 2007, Australia

^2^ ARC Centre of Excellence for Transformative Meta-Optical Systems, University of Technology Sydney, Ultimo, New South Wales 2007, Australia

^3^ Research Center for Electronic and Optical Materials, National Institute for Materials Science, 1-1 Namiki, Tsukuba, 305-0044, Japan

^4^ Research Center for Materials Nanoarchitectonics, National Institute for Materials Science, 1-1 Namiki, Tsukuba, 305-0044, Japan

**PL map of the emitter in Figure 1**

Figure 1S shows the confocal PL map at room temperature (23 ℃), under a magnetic field of ~73 mT. The yellow dotted line illustrates the edge of the hBN flake. The bright spot marked by the red circle is the quantum emitter with a narrow emission peak, shown in Figure 1(d).

*Figure S1. A Normalized PL map showing the hBN flake edge marked with yellow, and an emitter marked by red.*

**Simulation of the magnetic field in Figure 1(f)**

Figure S2 shows the simulated magnetic field intensity as a function of the distance from the permanent magnet. The calculation is performed using cylindrical approximation implemented in Python. The center distance is set to the distance where the magnetic field intensity is approximately 53 mT. In the calculated distance range, the magnetic field difference is ~10.9mT, which corresponds to a 304 MHz shift in the ODMR spectrum. This result is consistent with our calculated magnetic field intensity using ODMR shift.

*Figure S2. Simulated magnetic field intensity as a function of the distance from the magnet.*

**Magnetic field measurement at 22°C**

From the emitter in Figure 1, the magnetic field response was recorded at 22°C, Figure S3. The ZPL position was insensitive to the magnetic field with a standard deviation of 0.027 nm. Over the range of applied magnetic fields, the microwave resonance frequency changed, supporting the conclusion from Figure 1(f)

*Figure S3. Room temperature magnetic field response. Change in ZPL position (purple) and in resonant frequency (cyan) as a function of an external magnetic field, recorded at 22*°C *on the heating block.*

**Optical properties of emitters A and B in Figure 2**

Figure S4 shows full emission spectra, second-order autocorrelation function (g^2^(τ)), and lifetime of Emitter A and B shown in Figure 2 in the main text. Figure S4(a) shows the emission spectrum from Emitter A. For g^2^(τ) measurement, the emission was filtered from 600 nm to 620 nm (shaded area). The contribution of the target peak at ~610 nm to the measured g^2^(τ) is calculated by the ratio of the signal intensity to the total emission. The calculation is based on the four Gaussian function models (one for signal (filled area), the other three for background (dashed lines). Based on this calculation, the ratio of the signal intensity over the total emission spectrum within the filtered region is estimated to be 0.55.

Figure S4(b) shows the g^2^(τ) for Emitter A. As we did not observe the antibunching trend, we used a two-level system model as a fitting curve. Raw dataset was fit using the expression:

$g^{(2)}(\tau)=A\left( 1-p \cdot e^{-\frac{\left| \tau-\tau_{0} \right|}{\tau_{1}}} \right)$	(1)

Where *A* is amplitude factor, *p* is single photon purity (visibility of the antibunching dip), *τ_0_* is a horizontal offset correcting for timing mismatch, and *τ_1_* corresponds to the excited-state lifetime of the emitter. From the fitting, g^(2)^(0)=0.71 was extracted, shown as the dashed line in Figure S4(b). To subtract the effect of the background, we evaluated g^(2)^(0) using the BG-corrected g_cor_^(2)^(τ) function given by

$${g_{cor}}^{(2)}(\tau)=\frac{g^{(2)}(\tau)-(1-\rho^{2})}{\rho^{2}}$$

where *ρ = Signal / (Signal + BG)* is the ratio of the signal to the entire emission extracted from the emission spectrum. After background correction, g^(2)^(0) = 0.05 is extracted. This indicates that the temperature sensing is mainly contributed by the single emitters. Figure S4(c) shows the time decay of the emission intensity. To avoid the effect of the instrumental response function (IRF) shown by the purple dot line, we selected the data from 2 ns to 20 ns to fit. By the single exponential function fitting, the lifetime is extracted to 3.2 ns.

Figure S4(d), (e), and (f) exhibited the spectrum, g^2^(τ), lifetime for Emitter B, respectively. We note that the g^2^(0)=0.37 shown in Figure S4(e) is extracted without any background correction. From the time decay of the emission intensity, the lifetime is extracted to be 2.3 ns. These optical characteristics indicated the single-emitter natures of Emitters A and B, used for the localized sensing.

*Figure S4: Optical properties of Emitter A and B used for localized field sensing in the main text. (a) Emission spectrum from Emitter A. The shaded area indicates the filtered region for* *g^2^(τ) measurement. The black curve is a fitting curve using four Gaussian models. (b)* *g^2^(τ) function from the filtered region. The dashed curve is a fitting curve without background correction. The solid curve is a fitting curve with background correction based on the model shown in (a). (c) Decay curve of the emission intensity from Emitter A. The red dots show the data from the emitter and the purple dashed line corresponds to the IRF. The black line is a fitting curve of a single exponential function. (d) Emission spectrum from Emitter B. (e) g^2^(τ) function from the entire spectrum shown in (d). A dashed curve is a fitting curve without background correction. (f) Decay curve of the emission intensity from Emitter B. The orange dots show the data from the emitter and the purple dashed line corresponds to the IRF.*

**Comparison of experimental data in Figure 4 and simulation data in Figure 3**

Figure S5(a) and (b) shows the comparison of measured temperature (dots) and scaled simulated temperatures (dashed line) for Emitters A and B, respectively. The scaling factor is calculated by the comparison of the average temperature of measured and simulated values. The scaling factors are 0.54 for Emitter A and 0.49 for Emitter B. The close agreement between these values indicates that the discrepancy arises from a global systematic effect rather than position-dependent errors.

After applying the scaling, the simulated results are in agreement with the experimental temperature changes overall. A residual deviation remaining in the low-voltage regime is likely attributable to the neglect of the temperature dependence of the circuit's resistivity in the simulation.

Figure S5(c) and (d) represent the comparison of measured ODMR resonant frequency (dots) and simulated resonant frequency (dashed line) of Emitters A and B, respectively. The measured ODMR resonant frequency and simulated resonant frequency are good agreement. These agreements indicate that our sensing scheme correctly tracks the temperature and magnetic field changes.

*Figure S5: Comparison of the experimental results and simulated results for the microcircuit sensing. (a), (b) Comparison of measured temperature and scaled simulated temperature of Emitters A (a) and B (b). (c), (d) Comparison of measured ODMR resonant frequency and simulated resonant frequency of Emitters A (c) and B (d).*

**ODMR vs applied voltage and ZPL position vs magnetic field**

In Figure 4, Emitters A and B have a ZPL that was sensitive to temperature, and the resonance frequency was sensitive to the magnetic field, the corresponding plots for insensitivity are shown in Figure S6. Over the applied voltage range of 0 to 3.3 V and the resulting local temperature, the resonant frequency and the standard deviation for Emitters A and B were ~2.9 MHz and ~1.8 MHz. Over the applied magnetic field range of 63 to 79 mT, the ZPL peak position had a standard deviation of ~0.068 nm and ~0.058 nm for Emitters A and B, respectively.

*Figure S6. Two emitter insensitivity. (a) ODMR spectra for Emitters A and B over a voltage range of 0 to 3.3 V. The average resonant frequency was ~1.883 and ~1.886GHz, respectively. (b) Emission spectra for Emitters A and B recorded at different magnetic field strengths. The average ZPL position was ~610.0 and ~578.7 nm, respectively.*
